# Systematic identification of the role of gut microbiota in mental disorders: a TwinsUK cohort study

**DOI:** 10.1038/s41598-024-53929-w

**Published:** 2024-02-13

**Authors:** Julie Delanote, Alejandro Correa Rojo, Philippa M. Wells, Claire J. Steves, Gökhan Ertaylan

**Affiliations:** 1 Sustainable Health, Flemish Institute for Technological Research (VITO), Mol, Belgium; 2 Data Science Institute, Interuniversity Institute for Biostatistics and Statistical Bioinformatics (I-BioStat), Hasselt University, Diepenbeek, Belgium; 3 Department of Twin Research and Genetic Epidemiology, King’s College London, St Thomas’ Hospital, 3-4th Floor South Wing Block D, Westminster Bridge Road, London, SE1 7EH UK; 4 Department of Ageing and Health, St Thomas’ Hospital, 9th floor, North Wing, Westminster Bridge Road, London, SE1 7EH UK

**Keywords:** Microbiome, Predictive markers

## Abstract

Mental disorders are complex disorders influenced by multiple genetic, environmental, and biological factors. Specific microbiota imbalances seem to affect mental health status. However, the mechanisms by which microbiota disturbances impact the presence of depression, stress, anxiety, and eating disorders remain poorly understood. Currently, there are no robust biomarkers identified. We proposed a novel pyramid-layer design to accurately identify microbial/metabolomic signatures underlying mental disorders in the TwinsUK registry. Monozygotic and dizygotic twins discordant for mental disorders were screened, in a pairwise manner, for differentially abundant bacterial genera and circulating metabolites. In addition, multivariate analyses were performed, accounting for individual-level confounders. Our pyramid-layer study design allowed us to overcome the limitations of cross-sectional study designs with significant confounder effects and resulted in an association of the abundance of genus *Parabacteroides* with the diagnosis of mental disorders. Future research should explore the potential role of *Parabacteroides* as a mediator of mental health status. Our results indicate the potential role of the microbiome as a modifier in mental disorders that might contribute to the development of novel methodologies to assess personal risk and intervention strategies.

## Introduction

Neuropsychiatric disorders are complex conditions influenced by multiple factors, including genetic predisposition and environmental triggers^1–5^. Several studies have shown that patients diagnosed with a mental disorder (MD), including depression, anxiety, and eating disorders, demonstrate a dysbiosis of intestinal microbiota^6–9^. Specific microbiota imbalances affect the brain by humoral and neuronal mechanisms, with particular attention to the vagus nerve^10^. Gut microbiota can also have an impact through the endocrine pathway or by modification of the blood–brain barrier (BBB)^11^. Microbial metabolites mediators in this gut-brain signaling pathway, such as short-chain fatty acids (SCFAs), secondary bile acids, and amino acids, influence behavior, memory, learning, and locomotion^12^. In addition, clinical and preclinical evidence further support the existing gut-brain axis in neuropsychiatric disorders where fecal microbiota transplantation (FMT) from healthy donors has shown to be valuable in relieving depressive and anxious behaviors^13^, as well as the use of probiotics^14^.

Our microbiomes are inherently dynamic ecosystems that vary per individual. This complicates the identification of gut microbiota members that casually contribute to human disease. To truly identify microbial alteration associated with MDs, one must account for confounding factors, such as body mass index (BMI), dietary patterns, and antibiotic usage^15^. The maternal microbiota shapes the gut microbial composition, where perinatal factors are responsible for forming the adult-like microbiota^16^. However, there is still a wide interindividual heterogeneity in microbiota composition due to dietary traits, medication, lifestyle, and physiological variables^15^. Co-twin control study designs are effective in canceling off the majority of unmeasured differences. They offer a unique model for controlling confounders to interpersonal variation as they share genetic background, early-life events, and life course factors (e.g., birth delivery mode, feeding, dietary pattern, geography, etc.)^17^. Vilchez-Vargas et al.^18^ identified shared household and aging, *but not host genetics*, as factors in determining microbial similarity in fecal specimens of twins irrespective of zygosity. These findings emphasize the predominant role of environmental factors rather than host factors in defining the twins’ microbial composition^18^. In contrast, microbiome differences between participants, disease versus healthy, may be overpowered by interindividual variability in the microbiota in contemporary cross-sectional designs^15^.

Monozygotic (MZ) twins share all germline variants, whereas dizygotic (DZ) twins share, on average, half of their genetic material similar to non-twin siblings^19^. This study encompasses the analysis of depression, anxiety, and eating disorders as a collective group of MDs, which often co-occur and share common symptoms. The rationale behind this approach is to explore potential common impacts of the gut microbiota on these underlying disorders. Perturbations in the gut microbiome can influence various biological processes, including HPA axis dysregulation, immune system responses, mechanisms of inflammation, gut barrier permeability^20,21^, and alterations in the production of short-chain fatty acids and neurotransmitters^22,23^. These factors are integral to the proper functioning of neurological processes. By examining these disorders together, it is possible to uncover common patterns that contribute to the development and manifestation of these conditions. MDs were previously reported to have a considerable heritable component. Broad-sense heritability estimates for depression were reported in the range of 35–45%^24^; anxiety disorders between 35 and 50%^25^. For eating disorders, estimates are between 39 and 45% in binge eating disorder (BED), around 60% in bulimia nervosa (BN), and range from 28 to 74% in anorexia nervosa (AN)^26^. Assuming no somatic mutations occur, the comparison between MZ and DZ twins can be used to obtain insight into the degree of genetic involvement of a particular trait or disease^27^. If higher concordance rates are seen amongst MZ twins; compared to DZ twins, genetic factors might partially underlie the disease^27^. MZ twins were found to have higher concordance rates than DZ twins in the diagnosis of AN ^28–30^, BN^31,32^, anxiety and stress disorder^33,34^, and depression^35,36^. In light of this study, we posited that a desirable control is represented by the healthy co-twin of a discordant twin pair given the genetic relatedness to the MD-diagnosed twin and similar environmental exposures such as shared household as well as shared exposure from a maternal site like maternal breastmilk. By linking within-twin pair differences in mental health status to within-twin pair microbiome/metabolome differences, the discordant twin method has the unique potential to identify probable modifiable pathways between the twin’s mental health and microbiome/metabolome composition^37,38^.

Microbial alterations must be considered in light of their functional potential. It has been demonstrated that core housekeeping pathways remain stable across human microbiomes, such as ribosome and translational machinery, ATP synthesis, and glycolysis^39^. However, the unique microbial gene content translates to a huge diversity in how microbes produce or modify metabolites^40^. Seven times more associations were found between metabolites and microbial metabolic pathways than species, suggesting that functions rather than taxonomy reflect better the interplay between the microbiome and systemic environments^41^. Therefore, to benefit the host, modulations of the gut microbiome must target functionally related microbial communities rather than single microbes.

**Figure 1:**
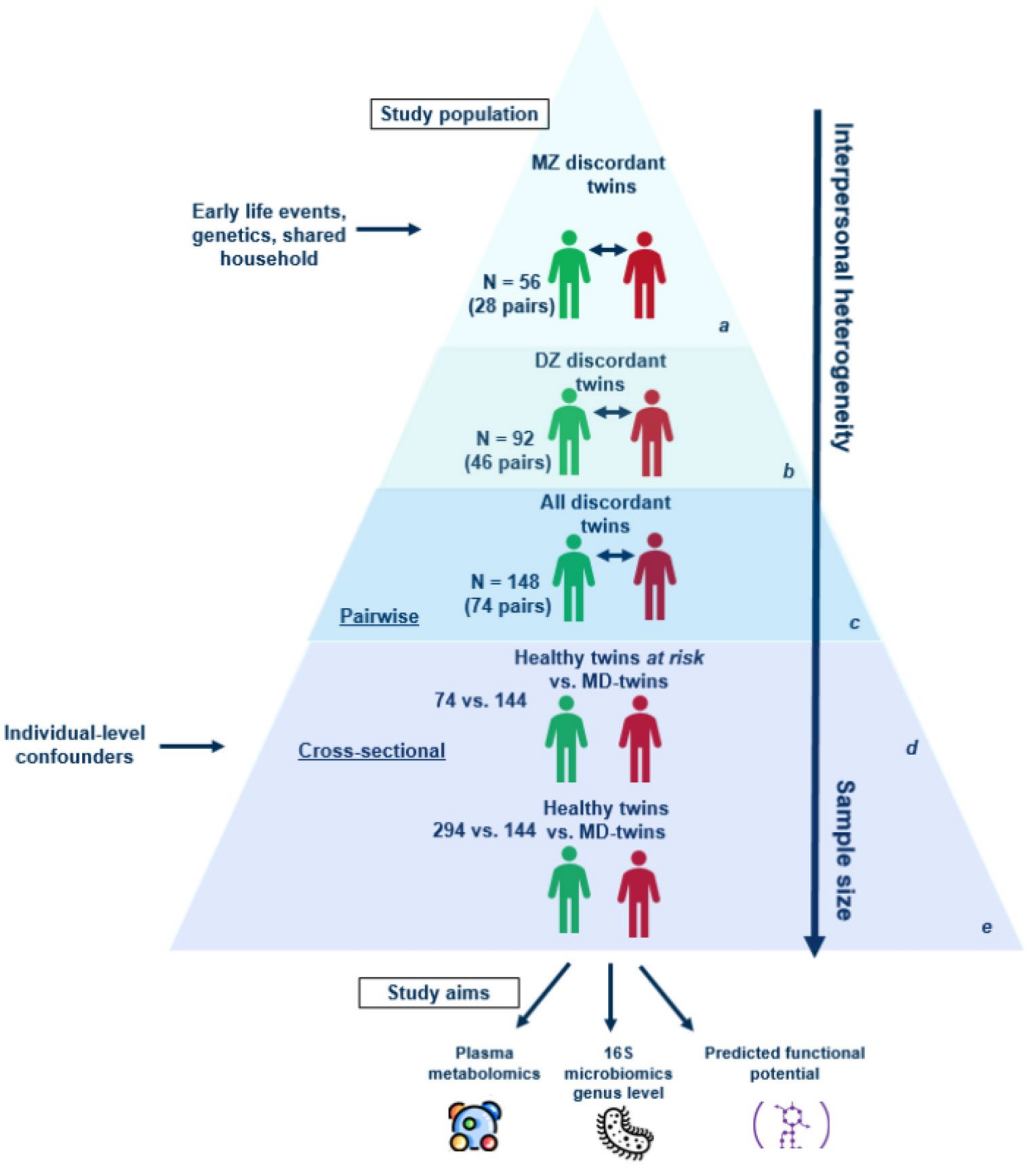
Visualization of the study design. The pyramid layers represent the different setups of this study. There are two main layers: (i) top layer: pairwise analyses in which we control for early life events, genetic background, and shared environment/household in which the twins grow up (ii) bottom layer: linear mixed regression analyses in which individual-level confounders were controlled for (antibiotics, diet differences, BMI, age). The top layer is divided into three parts (a) analysis of MZ twins, discordant in MD for a total of 28 twin pairs (b) analysis of DZ twins, discordant in MD for a total of 46 pairs, (c) analysis of twins, discordant for MD for a total of 74 pairs. The bottom layer studies (d) the healthy co-twins (who are assumed to be at increased risk of developing MD) with MD twins (a comparison of 74–144 twins) and (e) all healthy twins compared to all twins with MD diagnosis. The arrow indicates a higher interindividual heterogeneity and an increase in sample size the more you go toward the bottom of the pyramid. The study aims to find alterations in the microbiome and metabolome landscape in MD.

The gut microbiome plays an important role in mental health. A small number of research studies have identified potential microbiome-brain interactions implicated in several psychiatric conditions^42–44^. However, to our knowledge, there has been no single study that addresses gut (microbiome)-brain (function) interaction in twins’ settings. We hypothesized that the gut microbiome of healthy co-twins would differ from genetically similar twins with an MD diagnosis. Therefore, in this study, we characterized fecal microbiomes and plasma metabolomes using longitudinal data from the UK Adult Twin Registry (TwinsUK). Our aim is to discover taxa, plasma metabolites, and microbial pathways that may influence the onset/progression of MDs in adult twins, including potential differences in the gut microbiome between MZ and DZ twins. We propose a pyramid (layered) design approach for multiple comparison testing to identify differentially abundant features between sub-populations of twin types. The general flow of this study is represented in Fig. 1. The idea behind this structure is that we start from a specific population (MZ population) where twins share all of their genetic material, while the next population (DZ population) twins share, on average, 50% of their genetic material. Both populations are analyzed separately. We later combine the data of both populations to obtain more statistical confidence in possible associations between microbial genera/metabolites and MD status. To compensate for heterogeneity in the bottom layers of the pyramid, we account for potential individual confounding factors through larger sample sizes. Individuals from MD-discordant twin pairs who are not diagnosed with MD are referred to as “*healthy co-twins*” and individuals from MD-discordant or MD-concordant twin pairs who are diagnosed with MD are referred to as “*MD-twins*”.

The co-twin control study setup allows, *by design*, to control for genetic diversity and shared (childhood) environmental factors as important confounding effects. To this end, pairwise tests were performed in the population of MZ (Fig. 1, layer a), DZ (Fig. 1, layer b), and all discordant twins (Fig. 1, layer c). This setup allows us to do a direct comparison between twins who share the same genetic background and early environmental exposure and reduces the effect of potential confounders. Between MZ twins (Fig. 1, layer a), differences are more likely to be caused by other than shared genetic factors, while those identified in the DZ population (Fig. 1, layer b) could be due to both genetic and environmental effects. In layer c (Fig. 1, layer c), we combine MZ and DZ twins to increase sample size and potentially identify genera/metabolites associated with MD status. With this approach, the identification of microbial genera provides insight into the potential role of environmental factors in shaping the gut microbiome and its link with MD. These findings are then verified for non-shared environmental confounders, including BMI, dietary variables, and antibiotics usage, following multivariate analysis by using linear mixed models (Fig. 1, layers d and e). Regression analyses with linear mixed models allow us to examine the relationship between multiple variables while accounting for the correlation structure between the two twin types. By including non-shared environmental confounders as covariates, we can determine the independent associations of microbial genus/metabolite with health status. Specifically, layers (d) and (e) represent different aspects of the study design. Layer (d) focuses on studying all healthy co-twins, and are assumed to be at an increased risk of developing MD. This layer specifically examines the co-twins of individuals who have been diagnosed with MD (discordant twin pairs) in relation to any twin who is diagnosed with MD (from both concordant and discordant pairs). Layer (e) involves a broader comparison between all healthy twins and all MD twins (both from discordant and concordant twin pairs). Thus, layer (e) allows for a more specific investigation of the unique characteristics associated with being a healthy co-twin and the potential factors that contribute to their increased risk of developing MD, whereas layer (e) encompasses the larger, broad population. This study is the first exploratory analysis to discover potential microbiome-host interactions in the role of MD from genetically identical or similar individuals hence aims to minimize the role of genetics in gut microbiome-neurological phenotype associations. We only analyzed individuals with matched and complete microbiome and metabolome datasets, comprising a total of 438 individuals (219 twin pairs). Due to the small sample size of our study, we report associations with a false discovery rate (FDR) cut-off below 0.2 and report our findings in the context of earlier studies.

## Results

### General description cohort

A total of 438 twins (219 twin pairs) were included in the study, amongst which 424 were female and 14 male twins. 97 twin pairs were MZ, 122 twin pairs were DZ, and all twin pairs were same-sex pairs. Descriptive cohort characteristics are found in Table 1.

**Table 1:** Cohort characteristics.

		TwinsUK cohort		
Age (years)	mean (sd)	64.89 (7.71)		
BMI (kg/m^2^)	mean (sd)	26.33 (4.72)		
BMI ≥ 25	n (%)	155 (35%)		
BMI ≥30	n (%)	94 (21%)		
Gender (female)	n (%)	424 (96.8%)		
Gender (male)	n (%)	14 (3.2%)		

		All	MZ	DZ
Diagnosis of mental disorder(s)	n (%)	144	72	72
Healthy controls	n (%)	294	172	122

		All	MZ	DZ
Discordant pairs	# pairs	74	28	46
Concordant pairs (both MD)	# pairs	35	22	13
Concordant pairs (both healthy)	# pairs	110	47	63
		Total pairs: 219		

In this study, there is a prevalence of affected twins in the MZ population of 37.1%, while 29.5% in the DZ population. We found proband-wise concordance rates (probability that healthy co-twin will also have a history of MD) being 61% among MZ twins and 36% in DZ twins. No significant relationship was found between zygosity and the presence of MD (p = 0.114), nor between zygosity and phenotype concordance (p = 0.06562). However, when considering all MD-diagnosed twins, a significant relationship was found between concordance of the phenotype and zygosity (p = 0.004591). A significant relationship was also found between MD diagnosis and zygosity when considering all concordant twins (p = 0.00513). These results suggest that genetic factors may partially explain the MD phenotype that runs in twins. Given these results, we hypothesize that the healthy co-twin is at an increased risk of developing an MD which raises questions on how the microbiome might act as a potential modifier of this risk. Why is the healthy co-twin still healthy? Can the microbiome composition along with its functional effects contribute to his/her health status?

### Microbiome and metabolome composition are significantly more similar in MZ twins compared to DZ twins

**Figure 2:**
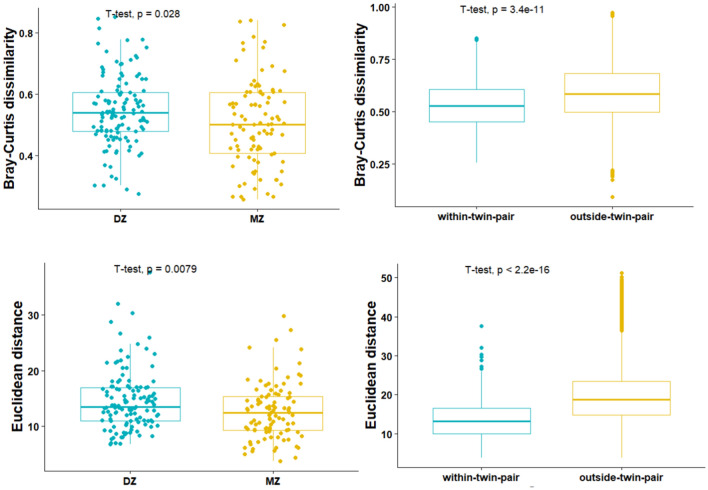
Microbiome and metabolome (dis)similarities between twins. Top: Bray–Curtis dissimilarity in DZ and MZ twins. within-twin pair refers to twin-twin dissimilarity in microbiome composition within a twin; outside-twin pair refers to each of the twins’ microbiome composition in comparison to each of the other twins (except his/her own co-twin). Bottom: Euclidean distances in DZ and MZ twins; and within-twin pair and between-twin analyses. Each dot represents a pair of twins (within-pair) or a pair of twins (independent of each other). Differences in dissimilarity were reported (Student’s t-test, significance level 0.05).

A comparison of Bray–Curtis dissimilarity metrics revealed a statistically significant greater dissimilarity in DZ twin pairs compared to MZ twin pairs (p = 0.028, Student’s t-test). Additionally, twins in the same twin pair demonstrated a significantly lower dissimilarity in their microbiomes, compared to twins from other pairs (p = 3.4e−11, Student’s t-test). Similar findings apply to the plasma metabolome composition, for which the Euclidean distance was used to compare metabolic profiles in MZ and DZ twin pairs. MZ twin pairs revealed a significantly lower Euclidean distance compared to DZ twin pairs (p = 0.036, Student’s t-test), as well as individuals in the same twin pair showed a significantly lower Euclidean distance compared to twins from other pairs (p < 2.2 e−16, Student’s t-test) (Fig. 2).

### Pairwise analyses of twins who are discordant for mental disorders

**Figure 3:**
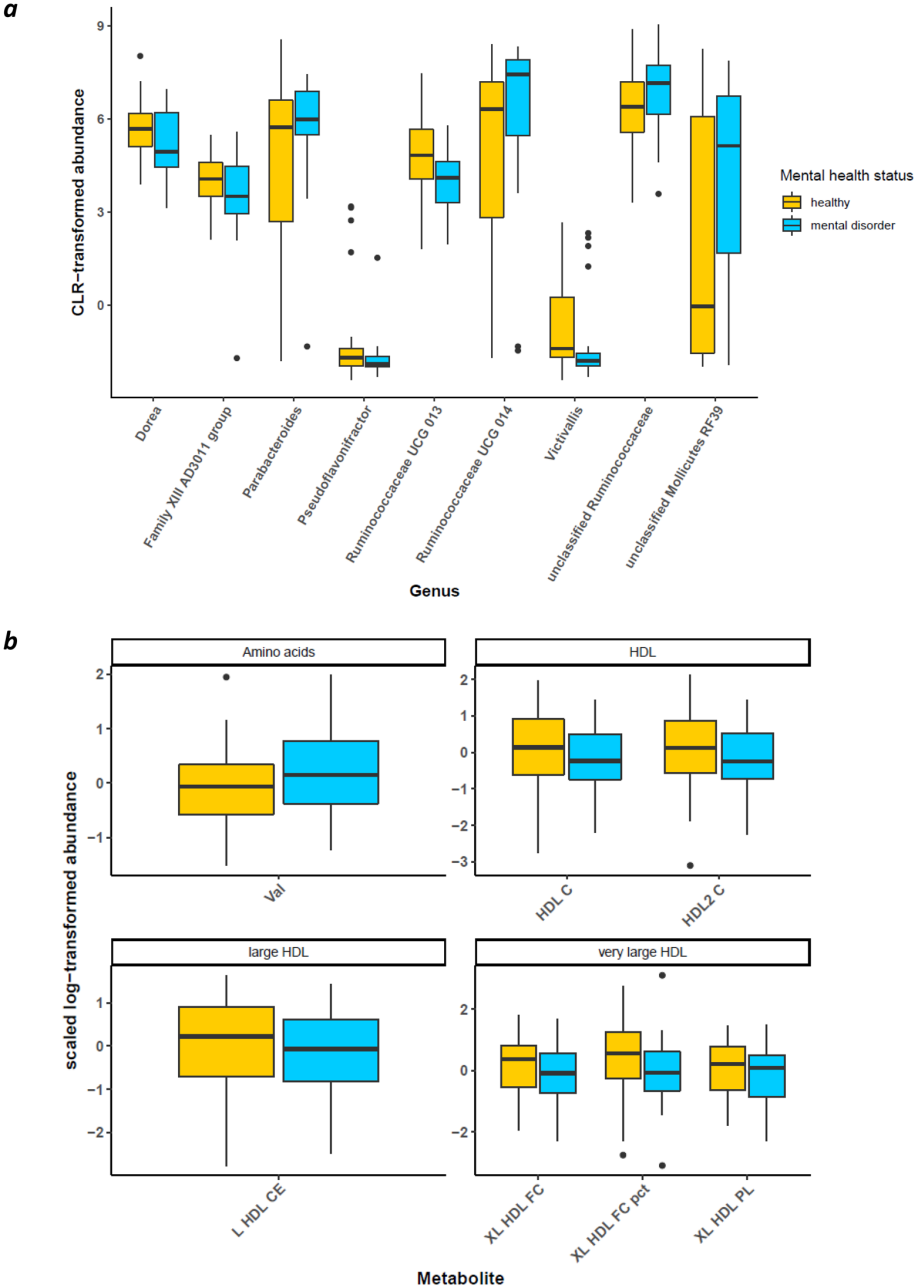
Pairwise analyses in the monozygotic twin population for microbiome and metabolome. (**a**) Significantly differentially abundant (p < 0.05, paired samples Wilcoxon test) bacterial genera identified in the MZ discordant analysis. (**b**) Significantly differentially abundant metabolites were identified in the MZ discordant analysis (same ordering as in (**a**)). Val, Valine; HDL-C, high-density lipoprotein (HDL) cholesterol; HDL2-C, HDL-2 cholesterol; L-HDL-CE, large HDL esterified cholesterol; XL-HDL-FC, very large HDL free cholesterol; XL-HDL-FC %, percentage very large HDL free cholesterol; XL-HDL-PL, very large HDL phospholipids.

A total of nine genera were found to be differentially abundant between MZ MD-twin and the healthy co-twin (MZ population, Fig. 1a), i.e., *Parabacteroides, Dorea, Ruminococcaceae UCG 014, Ruminococcaceae UCG 013, Family XIII AD3011 group, Victivallis, Pseudoflavonifractor,* unclassified *Ruminococcaceae,* and unclassified *Mollicutes RF39* (p < 0.05, paired samples Wilcoxon test) (Fig. 3a)*. *Metabolites that were significantly differentially abundant in MD-twins compared to healthy co-twins in the MZ population (p < 0.05, paired samples Wilcoxon test) included mainly high-density lipoprotein subfractions that were consistently lower in the MD group compared to the healthy co-twins. We found, in order of p-value, extra-large high-density lipoprotein free cholesterol (XL-HDL-FC %), Valine (Val), high-density lipoprotein 2 cholesterol (HDL2-C), XL HDL phospholipids, XL HDL free cholesterol, HDL cholesterol (HDL-C), large HDL particles (L-HDL-P), L HDL lipids (L-HDL-L), L-HDL cholesterol (L-HDL-C), L-HDL esterified cholesterol (L-HDL-CE) (Fig. 3b). Considering predicted metabolic pathways (Kyoto Encyclopedia of Genes and Genomes, hierarchical level 3), we found 13 predicted metabolic pathways to be significantly differentially abundant between MD and healthy co-twins (p < 0.1) (Supplementary Fig. S1).

The same pairwise analysis was performed in the population of DZ twins discordant in MD (DZ population, Fig. 1b). Out of the 18 significant genera identified, two genera overlapped with the findings in the MZ twin pairs, i.e., *Ruminococcaceae UCG 013* and *Family XIII AD3011 group* (Supplementary Fig. S2). Moreover, 60 metabolites were identified to be significantly different between MD and healthy twins (p < 0.05), but none of the ten metabolites identified in the MZ twin pair analysis were re-identified in the DZ twin pair analysis (Supplementary Fig. S3). The predicted metabolic pathway, folate biosynthesis, was identified as significantly differentially abundant between MD and healthy co-twins (p < 0.05). Other metabolic pathways involving vitamin metabolism were identified at a significance threshold of 0.1: ascorbate and aldarate metabolism (p = 0.09), and retinol metabolism (p = 0.09) (Supplementary Fig. S4).

**Figure 4:**
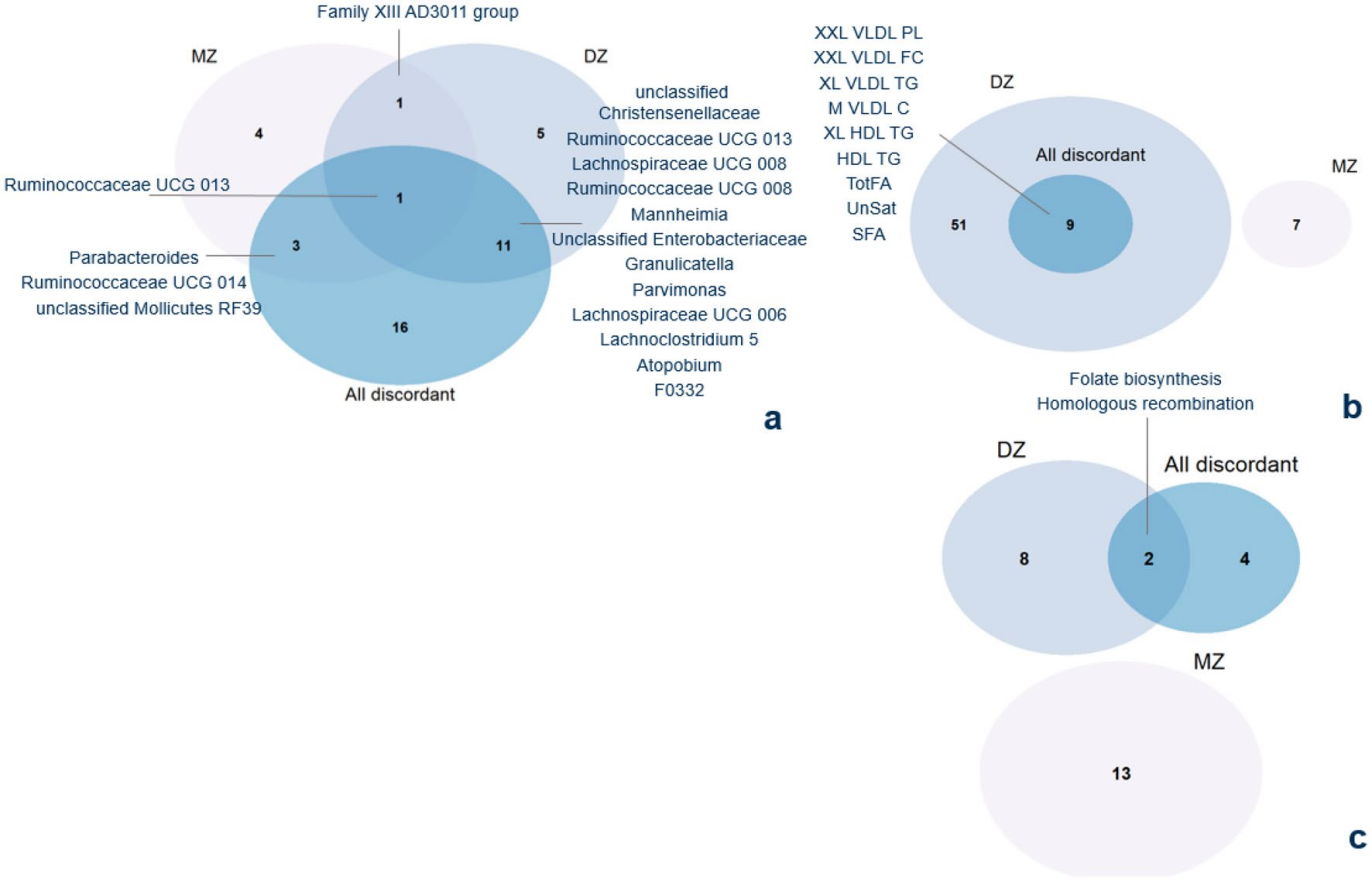
Venn diagrams representing overlaps between each of the pyramid layers: significantly identified microbes (**a**), metabolites (**b**), and predicted metabolic pathways (**c**). XXL-VLDL-PL, extremely large very-low-density lipoprotein phospholipids; FC, free cholesterol; TG, triglycerides; XL, very large; M, medium; HDL, high-density lipoprotein; TotFA, total fatty acids; UnSat, unsaturated fatty acids; SFA, saturated fatty acids; MZ, monozygotic; DZ, dizygotic.

When analyzing *all* discordant twins (combined MZ-DZ population, Fig. 1c), four of the genera identified in the MZ twin pair analysis, and 17 of the genera identified in the DZ twin pair analysis remained significantly abundant (p < 0.05). Regarding the metabolomics analyses, ten metabolites that were identified amongst the MZ twins could not be re-identified, whereas nine metabolites of the DZ twin pair analysis were significantly re-identified. Two metabolic pathways were identified in common between the pairwise analyses in DZ and all discordant twins: folate biosynthesis and homologous recombination. A summary of the overlapping findings is shown in Fig. 4.

### A distinct set of bacterial genera were found to be significantly associated with the diagnosis of mental disorder in linear mixed regression analyses

**Figure 5:**
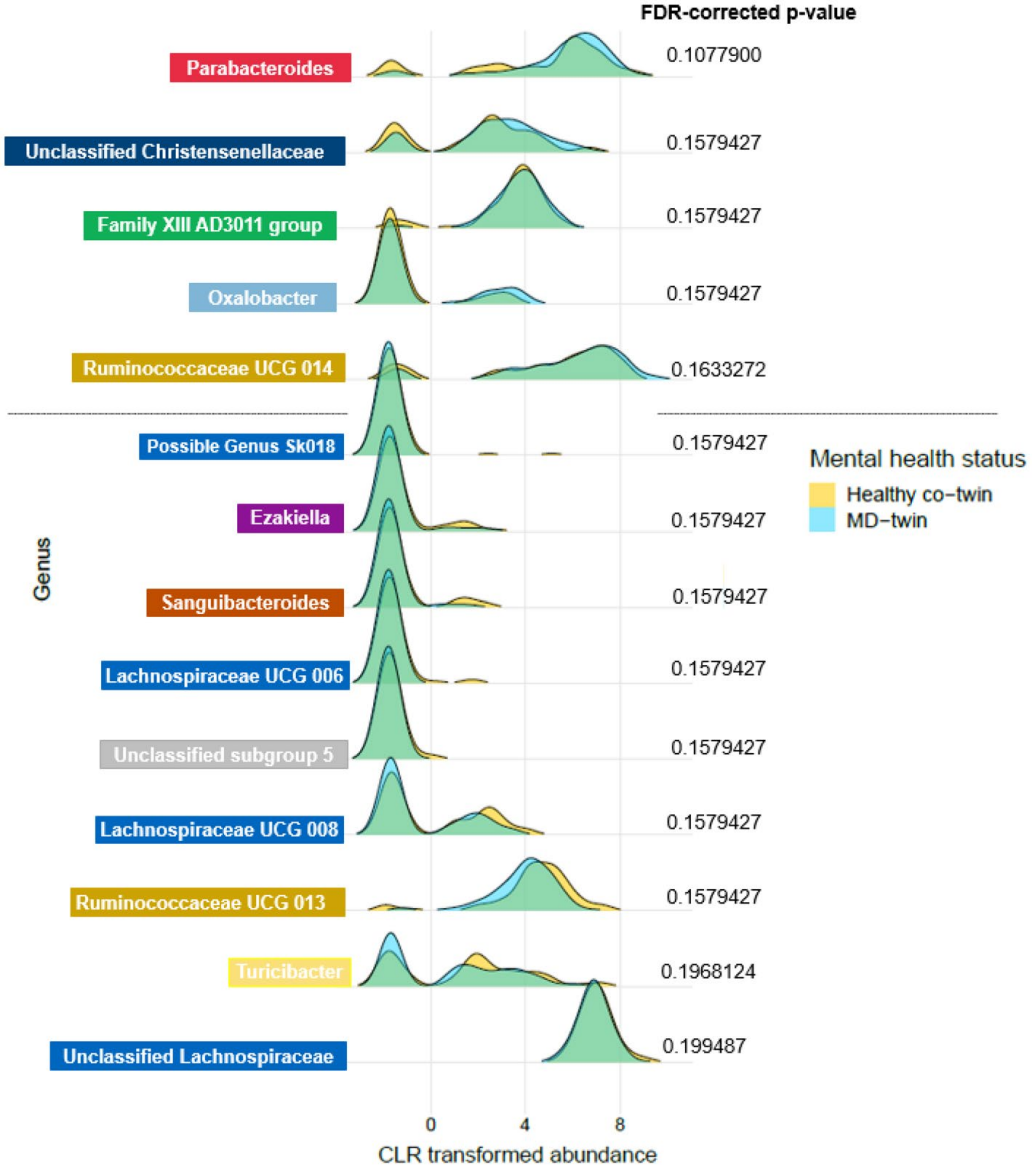
Distributions shown for genera significantly associated with MD-diagnosis in setup healthy co-twin (74) vs. MD-twins (144, all). Multiple testing corrections were performed using the Benjamini–Hochberg method and an FDR value < 0.2 was considered significant. Only significant genera are shown. The grey line separates those genera positively associated (top) and negatively associated with MD diagnosis (bottom). Genera in boxes with the same color are belonging to the same family.

A distinct set of associated bacterial genera and metabolites, resulting from previous analyses (Fig. 4), were regressed against individual-level confounders by using linear mixed models (LMMs) in two setups: healthy co-twins versus all MD-twins (risk-bearing, healthy co-twins vs MD twins, Fig. 1d), and all healthy twins versus all MD-twins (all healthy vs all MD, Fig. 1e). Confounders such as age, gender, BMI, antibiotics usage of the prior month, and vegetable and fruit intake were included. The MD diagnosis was used as a fixed effect, and random slopes were adopted to capture the twin’s zygosity. Out of the 41 bacterial genera, 14 genera were significantly associated with the MD diagnosis in a comparison of healthy co-twins (74) with MD-twins (144) (FDR < 0.2) (Fig. 5). Comparing their distributions, shifts can be seen in the more abundant taxa (left-skewed distributions with fewer negative CLR-transformed values), including genera Parabacteroides, Ruminococcaceae UCG 014, Ruminococcaceae UCG 013, Family XIII AD3011 group and unclassified Christensenellaceae. The existing imbalance in the microbial ecosystem coincides with shifts of more abundant taxa, along with multiple shifts in lower abundant taxa. This was not the case for the comparative setup of all healthy twins (294) with MD twins (144).

Predicted metabolic pathways (KEGG Level 3) from gut microbiome resulting from previous pairwise analyses were regressed against the same set of confounders and the fixed effect used for MD-diagnosis in LMMs. Metabolic functions of the MD gut microbiome were predicted to have roles in ascorbate and aldarate metabolism, retinol metabolism, metabolism of xenobiotics by cytochrome P450, drug metabolism by cytochrome P450, and microbial metabolism in diverse environments. On the other hand, non-MD gut microbiome metabolic functions involved pathways such as mismatch repair, carbapenem biosynthesis, glucagon signaling pathway, and homologous recombination (FDR < 0.25) (Supplementary Table S1). Regarding the metabolites significantly identified in previous analyses (Fig. 1a–c), none were significantly associated with MD diagnosis in either of the setups.

### Multivariate analyses

**Figure 6:**
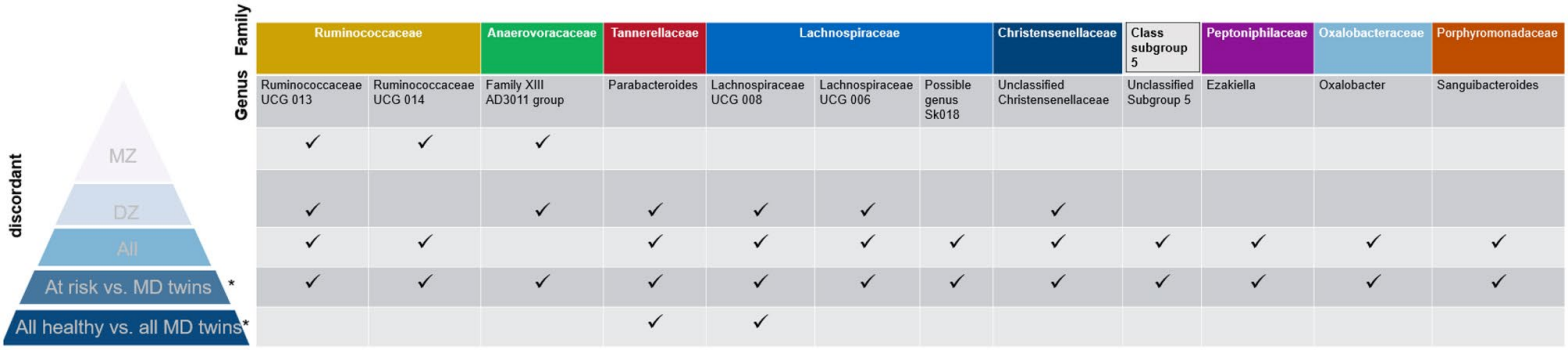
Pyramid layers complemented with significantly associated bacterial genera. Ticks (✔) indicate whether the bacterial genus was significantly identified in a particular layer of the pyramid. Genera are categorized per family represented by distinct colors (except for unclassified subgroup 5, which is identified up to the class level). MZ, monozygotic; DZ, dizygotic; MD, mental disorder; *: genera in these layers were significantly identified at a p-value threshold of 0.05, though no longer significantly identified after FDR correction.

As indicated in Fig. 1d, e, we compared healthy co-twins (n = 74) with all MD twins (n = 144) (Fig. 1d), followed by an analysis in which all healthy twins (294) were compared to all MD twins (144) considering **all** genera and **all** metabolites (Fig. 1e). Resulting from analyses in layer (d) we found, overlapping with previous analyses, genus *Parabacteroides* (increased), *Ruminococcaceae UCG 013* (decreased), *Family XIII AD3011 group* (increased)*, Ruminococcaceae UCG 014* (increased)*, Lachnospiraceae UCG 006* (decreased)*, Lachnospiraceae UCG 008* (decreased)*, Oxalobacter* (increased)*, Sanguibacteroides* (decreased)*, unclassified Christensenellaceae* (increased) and *Subgroup 5 *to be significantly associated with MD diagnosis (p-value < 0.05), after accounting for confounders gender, age, antibiotics usage of the prior month, vegetable and fruit intake. However, after performing FDR correction, these appeared no longer significant (FDR < 0.2) (Supplementary Table S2). Metabolites XL HDL TG, total FA, and saturated FA were identified to be significantly associated with MD diagnosis (p-value < 0.05) but remained no longer significant after FDR correction (FDR < 0.20) (Supplementary Table S3). Regarding analyses from **layer (e)**, genus *Parabacteroides,* and *Lachnospiraceae UCG 008* popped up to be significantly associated with MD diagnosis (P-value < 0.05), after accounting for confounders, though no longer significant after FDR-correction (FDR < 0.2) (Supplementary Table S4). None of the previously identified metabolites were found to be significant (Supplementary Table S5). Results of the microbiome analyses across the pyramid (Fig. 1) indicated that genus *Parabacteroides* was strongly associated with MD diagnosis (lowest p-value, highest estimate in LMM), though a stronger effect was seen in the comparison of healthy twins, at risk, in comparison with MD-twins. All bacterial genera that were significantly identified across the multiple layers of the pyramid are visualized in Fig. 6. Of the previously identified metabolic pathways, ascorbate and aldarate metabolism (increased), carbapenem biosynthesis (decreased), retinol metabolism (increased), drug metabolism by cytochrome P450 (increased), metabolism of xenobiotics by cytochrome P450 (increased) and homologous recombination (decreased) were re-identified in the setup of Fig. 1d (Supplementary Table S6). None of them were re-identified in the setup of Fig. 1e (Supplementary Table S7).

### Gut microbiota composition and functional profiles.

**Figure 7:**
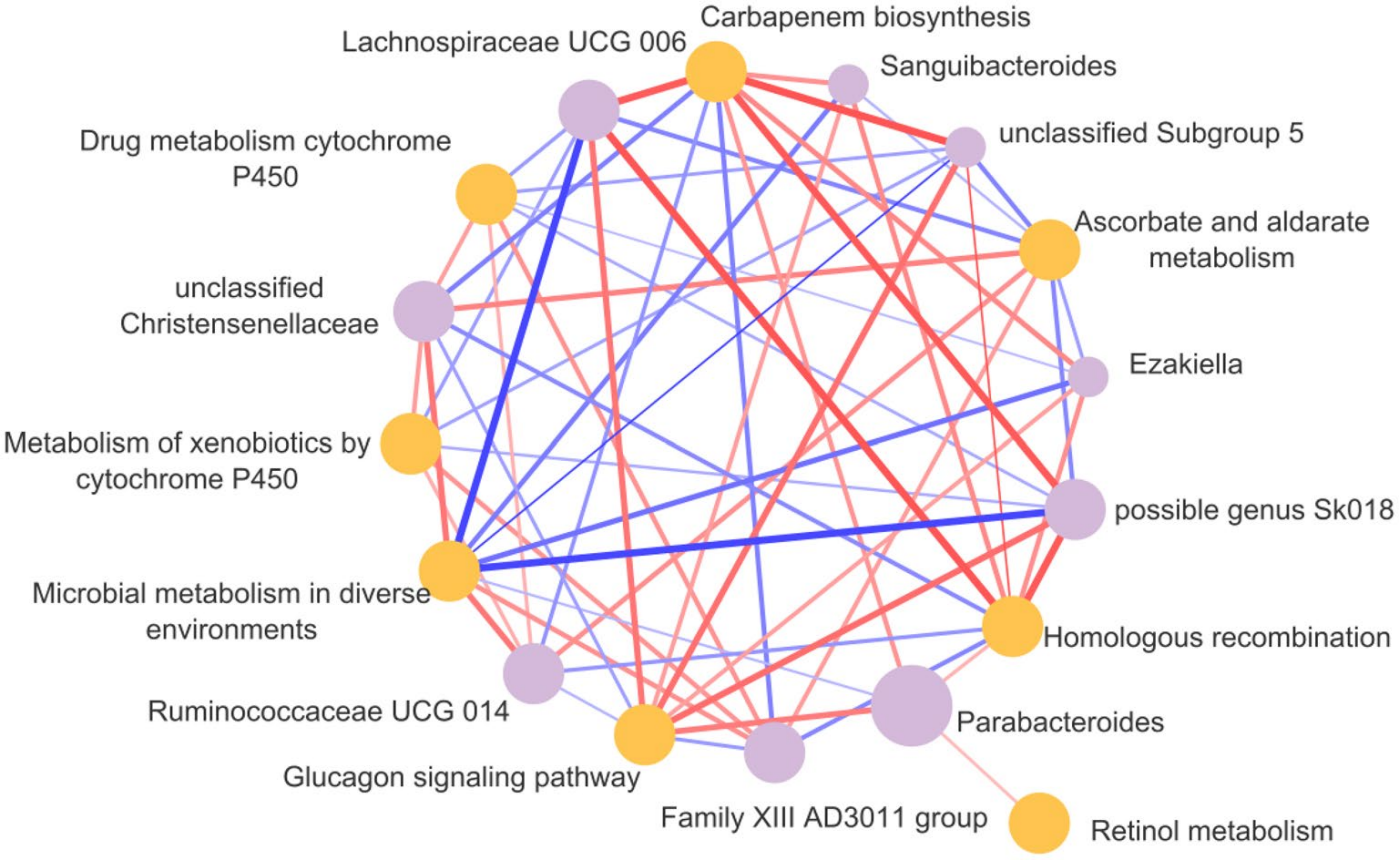
Correlation networks showings the relationships between differential microbial genera and the differential microbial KEGG level 3 metabolic pathways. The node sizes and colors (purple: differential microbial genera; orange: differential microbial metabolic pathways) are proportional to the number of significant associations across the layers of the pyramid. The width and color of the lines (red: positive, blue: negative) are proportional to the correlation strength. Only significant correlations (FDR-adjusted p-values with Benjamini–Hochberg procedure, < 0.05) are displayed, with a correlation strength >|0.2|. Permission has been obtained from Kanehisa laboratories for using KEGG pathway database^80^.

A correlation analysis using Spearman’s rank correlation coefficients revealed various significant correlations between previously identified differential abundant microbial genera and differentially abundant predicted metabolic pathways (Tax4Fun analyses). The correlation analysis was performed in the population of healthy co-twins versus MD-twins (Fig. 1d). A total of 24 correlations were identified (coefficient >|0.2|, FDR < 0.05). The top five correlations were related to genus Lachnospiraceae UCG 006: “ko00332: Carbapenem biosynthesis” (ρ = 0.54, p < 0.05), “ko03440: Homologous recombination” (ρ = 0.55, p < 0.05), “ko04922: Glucagon signaling pathway” (ρ = 0.45, p < 0.05). Moreover, unclassified subgroup 5 was significantly correlated with the “ko03440: Homologous recombination” (ρ = 0.57, p < 0.05) and “ko04922: Glucagon signaling pathway” (ρ = 0.47, p < 0.05). A summary of the correlation analysis is shown in Fig. 7.

## Discussion

Gut microbiome dysbiosis has been suggested to be a hallmark of mental disorders^45^. In our study, we profiled the gut microbiome and plasma metabolome in both MZ and DZ twins with MD-diagnosed and unaffected twins. Since MDs share genetic underpinnings, healthy co-twins were considered to be at an increased risk of developing an MD. We observed significantly higher similarity in gut microbiome and plasma metabolome composition in MZ twins, compared to DZ twins. This suggests that both microbiome and metabolome composition is shaped by experiencing similar environments, yet hinting at a host genetic effect. These findings support the co-twin control design, in which we control for both genetic diversity and early-life factors/environment that contribute to within-twin microbiome similarity.

Studying the gut microbiome in MD-discordant and concordant twin pairs aims to elucidate the contribution of the gut microbiota in the (risk of) MD development, because of the shared (childhood) environment, i.e., maternal sites and shared household as well as genetic factors between individuals from the same twin pair. We adopted a pyramid-layer design for our analyses. First, twins with the same zygosity were compared for MD in a pairwise manner. Second, healthy co-twins (healthy twins at risk) were compared with all MD twins, as well as *all* healthy twins against all MD twins. The latter, due to its higher sample size, increased statistical power and allowed us to validate the robustness of previous findings. These results allowed us to find candidate alterations in the gut microbiome or plasma metabolome landscape, assuming a shared genetic background and (childhood) environment. However, since these candidate alterations might as well be the result of individual-level confounders such as antibiotics usage, dietary influences, BMI, and age differences, they were validated in a multivariate design. There, we made a separation between two layers: (1) healthy cotwins in comparison to all MD twins and (2) all healthy versus all MD twins. This separation helps to identify specific alterations that may be associated with the risk of developing MD on the one hand (layer d) and on the other hand, layer (e) aims to provide a broader, comprehensive assessment of healthy vs MD twins across the larger population. The bottom layer aids in confirming the significance and association of microbial genera/metabolites with the MD diagnosis. In this way, significant *candidate* alterations in both microbiome and plasma metabolome were considered potential biomarkers when their presence was consistently noticed in all layers of the pyramid. As a result, genus *Parabacteroides* was identified in all layers, as most strongly associated with the MD diagnosis rather than with other individual-level confounders.

The increased abundance of genus *Parabacteroides* (family *Tenerellaceae*) is in concordance with recently published reports in which the genus was found to be elevated in depressed subjects compared to healthy controls^46–48^. Since *Parabacteroides* has been considered to have health-promoting effects, i.e., the production of SCFAs, the elevation of the genus in depressed subjects has been suggested to be a compensatory mechanism^48^. *Parabacteroides* species have also been linked to the production of metabolites γ-aminobutyric acid (GABA)^49^ and indole^50^ of which dysfunctions in their signaling pathways are related to depression and anxiety ^51,52^. *Parabacteroides* enrichment was shown to alter gene expression in pathways associated with metabolic function, neurodegenerative diseases, and dopaminergic signaling^53^. Hence, the *Parabacteroides* genus has been associated in previous reports with both protective and pathogenic effects on human health^54,55^. Nevertheless, the exact role played by this bacteria in depressive, stress, and anxiety-like behaviors remains to be investigated.

We found a positive correlation between the abundance of *Parabacteroides* and the glucagon signaling pathway (Fig. 7). The role of *Parabacteroides* species in the context of metabolic dysfunctions has been discussed in several earlier reports, though with inconsistent findings.

As previously suggested, we would like to argue that functionally related microbial communities rather than single microbes have an overall impact. First, genus *Parabacteroides* has been confirmed in a recent meta-analysis to be enriched in major depressive disorder in more than 20% of the included studies (24 in total). The authors highlighted *Parabacteroides’* involvement in greater utilization of glutamate and increased synthesis of GABA^56^. In our research, we noticed overlapping distributions of this genus’ (and other genera) abundance in healthy co-twins compared with MD-twins, though with a slightly larger abundance in MD-twins (Fig. 5). However, due to the resolution of the TwinsUK dataset, the higher abundance of this specific genus cannot be brought back to specific species. Second, diagnoses of mental disorders in our research were not limited to depressive disorders, but also include anxiety, stress, and eating disorders that might explain the less strong association compared to earlier studies. For instance, a high abundance of Parabacteroides could be an indication of MD symptoms. Other genera that were identified through multiple layers from our pyramid design include decreased abundances of genera *Ruminococcaceae UCG 013, Lachnospiraceae UCG 008, Lachnospiraceae UCG 006,* and increased abundances of *Ruminococcaceae UCG 014, Family XIII AD3011 group,* and unclassified *Christensenellaceae*. Genera of families *Lachnospiraceae* and *Ruminococcaceae* were previously associated with the maintenance of gut health^57^. Members of *Lachnospiraceae* are among the main producers of SCFA, though different taxa of this family have been associated with intra- and extraintestinal diseases^58^. Since *Lachnospiraceae* are strongly influenced by diet specifics, such as non-starch polysaccharides and high-fat feeding^58^, the associations in both directions were not unexpected. In contrast to other reports, only a few genera were identified in significant association with MD diagnosis. Gut microbiome composition may be different in patients with an MD in partial or full remission compared to patients with a current illness phase. Since we could not account for illness states, state-related changes in the gut microbiome composition could not be established in our study design^59^. We suggest combining multiple omics approaches (meta-transcriptomics, proteomics, metabolomics) to reveal a *systems-level insight* into functional differences rather than to retain a compositional focus.

In this study, we observed that metabolic functions involving vitamin metabolism, including retinol and ascorbate and aldarate metabolism were more abundant in the gut microbiome of patients with MDs compared to non-MD controls. It has been hypothesized that vitamin A levels are inversely associated with depression^60,61^. In addition, previous work has established the role of gut microbiota in vitamin A (retinol) metabolism^62,63^. By regulating the expression of gene *Rdh7*, gut bacteria can curb the production of vitamin A metabolite retinoic acid (RA). The increase in ascorbate metabolism, associated with MD is, in contrast, to what has been found earlier^63^. However, the pathophysiological association of ascorbate with depression is currently not clear^64^.

In the setup of healthy co-twins, assumed to be at risk of developing MD, in comparison with MD-twins (Fig. 1d), more genera were found to be significantly associated with MD than when considering the setup of all healthy twins compared with the MD-twins (discordant *and* concordant twins included). In addition, the size of the estimate of *Parabacteroides* in association with MD diagnosis was stronger in the former setup. Thus, the inclusion of healthy concordant twins (layer e) (no identifiable risk of MD) weakened the association of *Parabacteroides* with MD diagnosis. The number of genera identified and consequently the metabolic pathways differentially identified were different between the two setups (Supplementary Tables S2, S4, S6–7). Further studies with increased study sample sizes are expected to unravel the contributions of microbiota in individuals at increased genetic risk. Unlike other omics data, the microbiome’s dynamic nature does not only suggest a role as *a modifier* to disease risk, but, not unimportantly, appears very suitable for real-life applications in terms of privacy and security reasons^65^.

Compared to healthy controls, none of the metabolomic alterations identified in the pairwise analyses (both MZ and DZ) remained significant after correcting for individual-level confounders. This stands in contrast to several reports that showed plasma metabolomic markers to discriminate healthy controls from individuals with depression and anxiety^66–69^. Plasma metabolites identified in pairwise analyses in the MZ group (Fig. 2a) varied greatly from those identified in the DZ group (Supplementary Fig. S3). First, the NMR platform mainly captures lipid metabolites rather than the full metabolome profile. Second, the variation might be explained by the BMI differences in the MZ and DZ population, between the MD-diagnosed and control group (Supplementary Fig. S5). The control group in the DZ population demonstrates a higher BMI, compared with the MZ population, along with higher levels of VLDL metabolites in the DZ control group and higher levels of HDL in the MZ control group. This is in line with previous reports on dyslipidemia and obesity^70,71^. To cope with this issue, future work should envision individual reference intervals, that exploit longitudinal subject-specific data rather than looking into population ranges^72^.

Our study has several limitations. First, while highly relevant covariates were included (age, antibiotics, gender, vegetable, and fruit intake), other environmental triggers might not have been comprehensibly tracked. Seen the major contribution of dietary influences on the host’s microbiome, more detailed dietary information on micro- and macronutrients and different food groups must be considered to yield better insights into the microbiome-metabolome-brain crosstalk. This will allow us to gain insight into what dietary patterns are protective and contribute to better mental health. Second, *all* individuals with diagnoses of MD were included, i.e., diagnoses that were ongoing at the moment of sample collection, as well as individuals previously diagnosed with an MD. When the patient is under treatment or has overcome his/her mental illness, the microbiome composition is expected to change again to restore gut dysbiosis^73^. Third, a more balanced population sampling exploiting additional cohorts is necessary to obtain more reliable results. The TwinsUK cohort consists of mainly female individuals (96%). Therefore, the research results are mainly representative of the female population. A gender-specific version of performed analyses might be considered to see the existing sex differences in behavior and risk for neuropsychiatric disorders^74^. Fourth, several diagnoses of MDs were categorized under the same heading to identify common pathways emerging in individuals with MDs, including depression, anxiety and stress, and eating disorders. Although the included diagnoses of disorders differed in their pathophysiology, common pathways are emerging in psychiatric diseases with immune dysregulation being present in a broad spectrum of neuropsychiatric disorders, increasing mental vulnerability and risk of psychiatric symptoms^75^. Furthermore, these diagnoses have shown associations with increased gut permeability, impaired gut-brain communication, and disrupted neurotransmitter production^20–23,76–78^. The combination of these factors highlights the intricate interplay between the gut microbiota, the immune system, and neurological processes in the context of MDs. Fifth, although the identified changes in the intestinal microbiota have been previously identified along with its mechanisms of altered glutamate and GABA metabolism and butyrate production^56^, we would like to emphasize the need for more established protocols in microbiome studies to reveal the relationships between mental disorders and the gut microbiome (Supplementary Information, Appendix 1). Importantly, the utilization of the 16S rRNA gene sequencing method, which is inherent to the TwinsUK study design, imposes certain limitations on the extent of the microbiome analysis. The method is unable to provide species-level resolution or detailed information on the functional capacity of the microbiota. However, we applied the method with the overall idea to identify differences in the structure and composition of microbial communities. Moreover, we agree that longitudinal cohort studies, the inclusion of confounding covariates to disentangle cause/consequence as well as the exploitation of multi-omics approaches will aid to uncover the activity of our microbiomes beyond their composition.

## Conclusions

In this study, we robustly identified that individuals diagnosed with MD showed a greater abundance of the genus *Parabacteroides*. This genus might offer a novel opportunity for primary prevention. However, the exact role and effect of this genus in MD initiation and progression remain to be investigated in further studies as the effects are likely species-specific. In general, we can conclude that our presented pyramid-layering design in a population of twins offers a robust manner to identify differentially abundant features in diseases with both genetic and environmental components underlying. We propose microbiome-mediated mechanisms as likely gut biomarkers and/or mediators in the context of MD as a rationale for future research.

## Materials and methods

### Study population

The TwinsUK cohort is a longitudinal dataset and the largest research cohort of adult twins from the United Kingdom (http://www.twinsuk.ac.uk/). The cohort comprises over 14,000 volunteers followed over more than two decades. Participants were predominantly female (> 80%) and aged 18–103. This cohort has been extensively studied for a wide range of clinical and behavioral outcomes. All work involving human subjects was approved by the Cornell University IRB (Protocol ID 1108002388) and all methods were performed following relevant guidelines and regulations. Informed consent was obtained from all participants.

**Figure 8:**
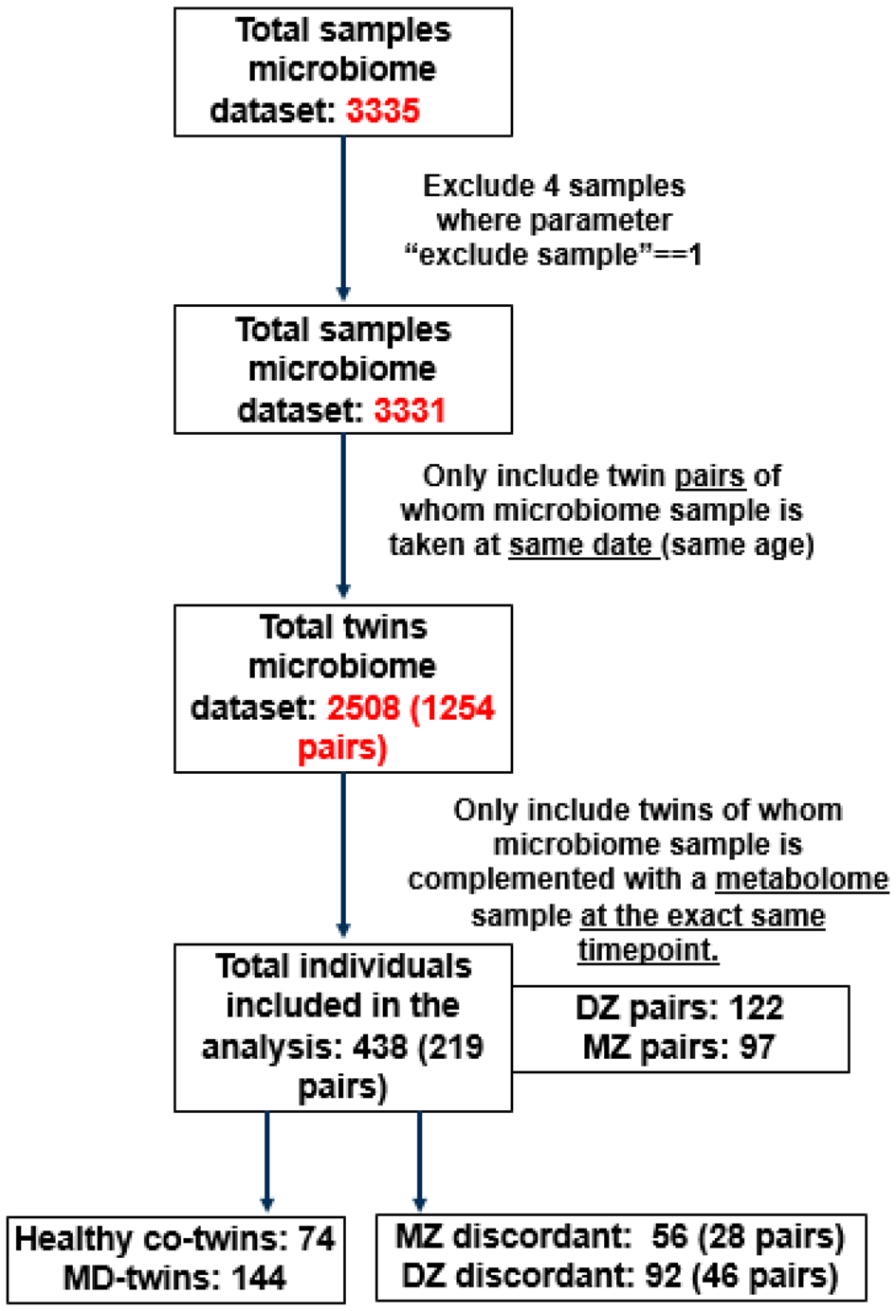
Overview of selected samples (microbiome/plasma metabolomics).

In our study, 438 twins (219 pairs) were included, who have both 16S rRNA gut microbiome data and concurrent plasma metabolomics data at the same time point. Amongst the 438 twins (219 twin pairs), 122 were DZ, and 97 were MZ twin pairs (Fig. 8). Regarding the phenotypic variable on MD, different groups were specified according to the Hospital Anxiety and Depression (HADS) questionnaire as follows. For participants that have one of the questions related to Depression (see Appendix 1) specified on “1” (yes), “Anxiety/Stress disorder” and “eating disorder”, the variable “MD” was specified on “1” (yes). Individuals who had none of the MD-related variables specified on “1”, were defined as “healthy control”, in the assumption that “healthy” means no diagnosed MDs. The final dataset consisted of 294 controls and 144 cases with one or more MDs diagnosed. Detailed description of the implementation of the *Hospital Anxiety and Depression (HADS)* questionnaire in TwinsUK cohort is available^79^.

Microbiome sequences from 3335 samples, including metabolic abundances from 4830 samples, were collected from the TwinsUK cohort. First, for all samples in the microbiome dataset, twin pairs were screened based on zygosity, age, year of birth, the time point at which the microbiome sample was taken, and type of descendant. Only pairs with microbiome samples matched with their corresponding time point were considered, resulting in 1254 pairs with 2508 samples. Next, from these microbiome samples, only 524 individuals were matched with their complementary metabolome samples at the same time point. Finally, only twin pairs without missing measurements across time points were considered in the analysis. The final dataset consisted of 438 individuals (219 twin pairs), with microbiome and metabolome measurements from 2010 to 2015.

### Data preprocessing

The microbiome data from the TwinsUK cohort was delivered in a preprocessed format, i.e. amplicon sequence variants (ASVs) were generated using the DADA2 pipeline. Samples achieving a sequence depth of less than 10,000 were excluded, and sequences that were unassigned at Kingdom/Phylum level were removed. Taxonomy was assigned to the ASV sequences with the SILVA reference database^80^. In the next step, the ASVs were imported, together with their associated metadata (see Metadata processing) using the *phyloseq* package (v1.40.0)^81^. The dataset was filtered using the following criteria: (1) taxa observed only once in the whole dataset were removed, (2) taxa must appear in more than one sample, (3) ASVs with no counts were removed (4) empty samples were removed (none in the dataset). Finally, data were agglomerated to genus level and transformed by using the centered log-ratio transformation (CLR), i.e. each of the taxon counts is divided by the geometric mean of all taxa in a sample, and subsequently logarithmically transformed. The CLR transformation was implemented in the *microbiome* R package (v1.18.0).

Metabolic measurements were available for 228 metabolic traits: 147 metabolite concentrations (comprising groups: Cholesterol, Lipoprotein subclasses, Glycerides and Phospholipids, Apolipoproteins, Total Fatty acids, Amino Acids, Ketone bodies, Fluid balance, and Inflammation), 77 lipid ratios, three lipoprotein particle sizes and a semi-quantitative measure of albumin. Metabolic profiling was conducted by Nightingale Health Ltd. (Helsinki, Finland) using a targeted NMR spectroscopy platform. These metabolic measurements were log-transformed; for all measurements before transformation, a pseudo-count of 1 was added. Next, measurements were shifted to zero mean and scaled to a standard deviation (SD) of 1^82^. Metabolites that were assigned missing values in more than 25% of the individuals were removed. Metabolites that had missing values, in less than 25% of the individuals, were replaced by their median value. For the confounder-related questionnaire data, selected features were included that are known to confound gut microbiome composition: age, gender, BMI, antibiotics, vegetable and fruit intake^83^. Data was taken at the time point at which the microbiome/metabolome sample was collected; if not available, data were collected from the closest available time point. Missing values were replaced by the median value of the respective variable.

### Statistical analyses

The statistical analyses were performed using the R statistical software version 4.1.2 (2021–11-01, https://www.rstudio.com/products/rstudio/download/), and different libraries included in Bioconductor (v3.13) were used, including *microbiome, vegan* (v2.6.2), and *phyloseq.* Microbiota beta-diversity was calculated by the Bray–Curtis dissimilarity metric, which quantifies the compositional dissimilarity between two samples or groups, considering both overall abundance per sample as well as the abundance of each taxon^84^. The Euclidean distance was used for comparisons of metabolic profiles. Tests for paired data were used to address the between-group comparisons (MD vs healthy) of continuous variables. The Shapiro–Wilk’s method was used to test for normality of the distribution and accordingly, the student’s t-test (p-value > 0.05) or Wilcoxon’s test was used. The level of significance was set at the two-tailed p-value of 0.05.

For the multivariate analyses, association analyses were performed for (1) healthy co-twins vs. MD-twins and (2) healthy twins vs. twins with MD. For this, a modified version of the model proposed by New et al.^85^ is proposed as follows:

$$Y_{i} \sim {\text{N}}(\alpha_{{Z_{j\left[ i \right]} + }} X\beta ,\sigma^{2})$$

$$\alpha_{MZ,j } \sim N \left( {0, \sigma_{MZ}^{2} } \right), for\, MZ\, twin\, pair\, j = 1, ... , J_{MZ}$$

$$\alpha_{DZ,j} \sim N\left( {0, \sigma_{DZ}^{2} } \right) , for\, DZ\, twin\, pair\, j = 1...., J_{DZ}$$

where ${Y}_{i}$ is the microbe/metabolite abundance; ${\alpha }_{{Z}_{j[i]}}$ is the random effect where accounted for either MZ or DZ twins, i.e. ${\alpha }_{MZ,j}$ and ${\alpha }_{DZ,j}$ respectively; $X$ are the predictor variable(s), $\beta$ are the slope estimates explaining the contribution of the predictor variable to the dependent variable, and ${\sigma }^{2}$ is the variance. In the model, each twin was assumed to have a random slope, specifically random slopes for MZ twins and for DZ twins coming from two different distributions. The model estimates variance (relatedness) between MZ twins separately from the variance between DZ twins (relatedness) between DZ twins. CLR-transformed bacterial genera abundances and scaled, log-transformed metabolite abundances were regressed against main confounders: age, gender, BMI, antibiotics usage prior month, vegetable and fruit intake. The regression was done by using a LMM, using the *nlme* R package (v3.1.157). The MD categorical variable was included as fixed effect. We corrected for multiple testing by using the Benjamini–Hochberg approach. For multivariate analyses, FDR-thresholds were relaxed to 0.25 since we are interested in an exploration and screening of potential features (bacteria/metabolites) of interest and thus less strict on the inflated type-I error.

Differentially abundant bacterial genera along with differentially abundant KEGG pathways, that were significantly associated with MD-diagnosis in the multivariate analyses, were used in the correlation analysis based on Spearman’s rank correlation coefficients. The cutoffs for the correlation coefficients and adjusted p-values (FDR correction with Benjamini–Hochberg procedure) were determined to equal 0.2 and 0.05, respectively. Networks were built using Cytoscape (https://cytoscape.org/; v3.9.1) to represent the correlation network of bacterial genera and metabolic pathways.

### Predicted functional potential

The Tax4Fun2 open-source R package (v1.1.5) was used to predict the functional capabilities of the microbial communities based on 16S rRNA datasets, applicable to SILVA-labeled microbial abundances^86^. To predict the community function, the table with sequences of ASVs and the table with sequence abundances were required. Both tables were read by Tax4Fun2 through an external file; sequences were saved in FASTA format through the write.fasta() function from the seqinr package. In the first step, the sequences of the samples were aligned with the package reference database via BLAST, using the runRefBlast() function with database_mode option “Ref100NR” for greater precision in alignment. Next, makeFunctionalPrediction() function was used to obtain the prediction of the abundance of functions in the community, with a similarity cut-off set to 97%. As a result, two files were generated. The first one contained KEGG ortholog profiles, i.e., predictions of the relative abundances of each enzyme indicated for each sample, according to their composition (functional predictions). The second one contained predictions on the abundances of the metabolic pathways of each sample according to its composition. The latter was organized into three hierarchical levels, with different categories as corresponding to the metabolic pathways in the Kyoto Encyclopedia of Genes and Genomes (KEGG)^87^. Tax4Fun2 output for the level 3 KEGG entries were analyzed.

### Ethics declarations

Ethics approval was granted by the St. Thomas’ Hospital Research Ethics Committee. Following the restructure and merging of the research ethics committee, subsequent amendments were approved by the National Research Ethics Service (NRES) Committee London–Westminster (TwinsUK reference EC04/015); approval for the use of microbiota samples was granted by the NRES Committee London– Westminster (The Flora Twin Study reference 12/LO/0227).

### Supplementary Information

Supplementary Information.

## Data Availability

The data that support the findings of this study are available from TwinsUK but restrictions apply to the availability of these data, which were used under license for the current study, and so are not publicly available. Data are however available from the authors upon reasonable request and with permission of TwinsUK. Accession codes: The European Bioinformatics Institute (EBI) accession numbers for the microbial DNA sequences reported in this paper is ERP015317.
