# Supplementary material for: Systematic identification of the role of gut microbiota in mental disorders: a TwinsUK cohort study
Source: Sci Rep. 2024 Feb 13;14:3626. doi: 10.1038/s41598-024-53929-w (PMC10864280; doi:10.1038/s41598-024-53929-w)

# Supplementary Figures

Title: Systematic identification of the role of gut microbiota in mental disorders: A TwinsUK cohort study

Julie Delanote, Alejandro Correa Rojo, Philippa M. Wells, Claire J. Steves*,* Gökhan Ertaylan


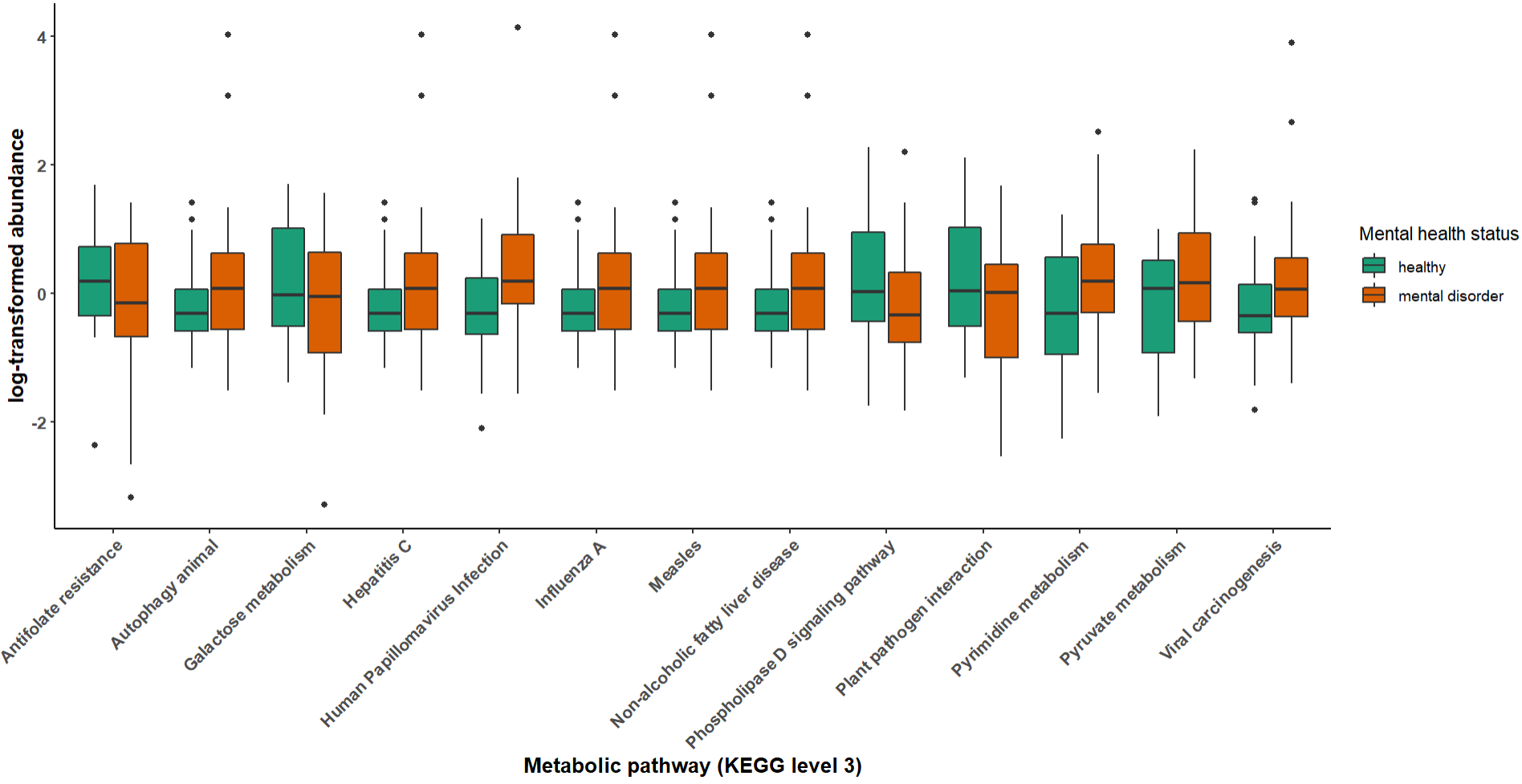


**Supplementary Figure S1** Predicted metabolic pathways (KEGG Level 3) differentially abundant in monozygotic discordant twins (paired samples Wilcoxon test, p-value <0.1).


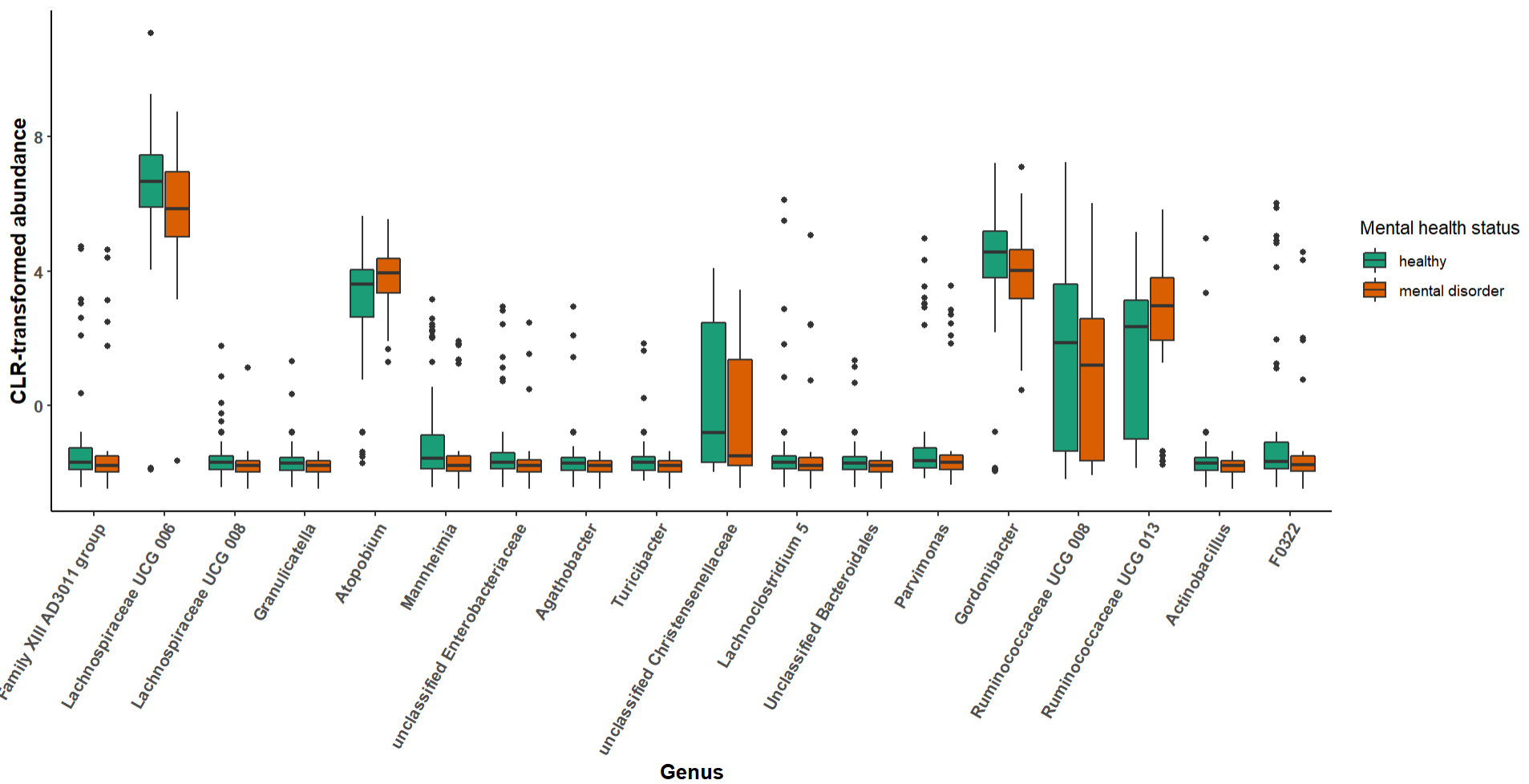


**Supplementary Figure S2** Genera differentially abundant in dizygotic discordant twins (paired samples Wilcoxon test, p-value <0.05).


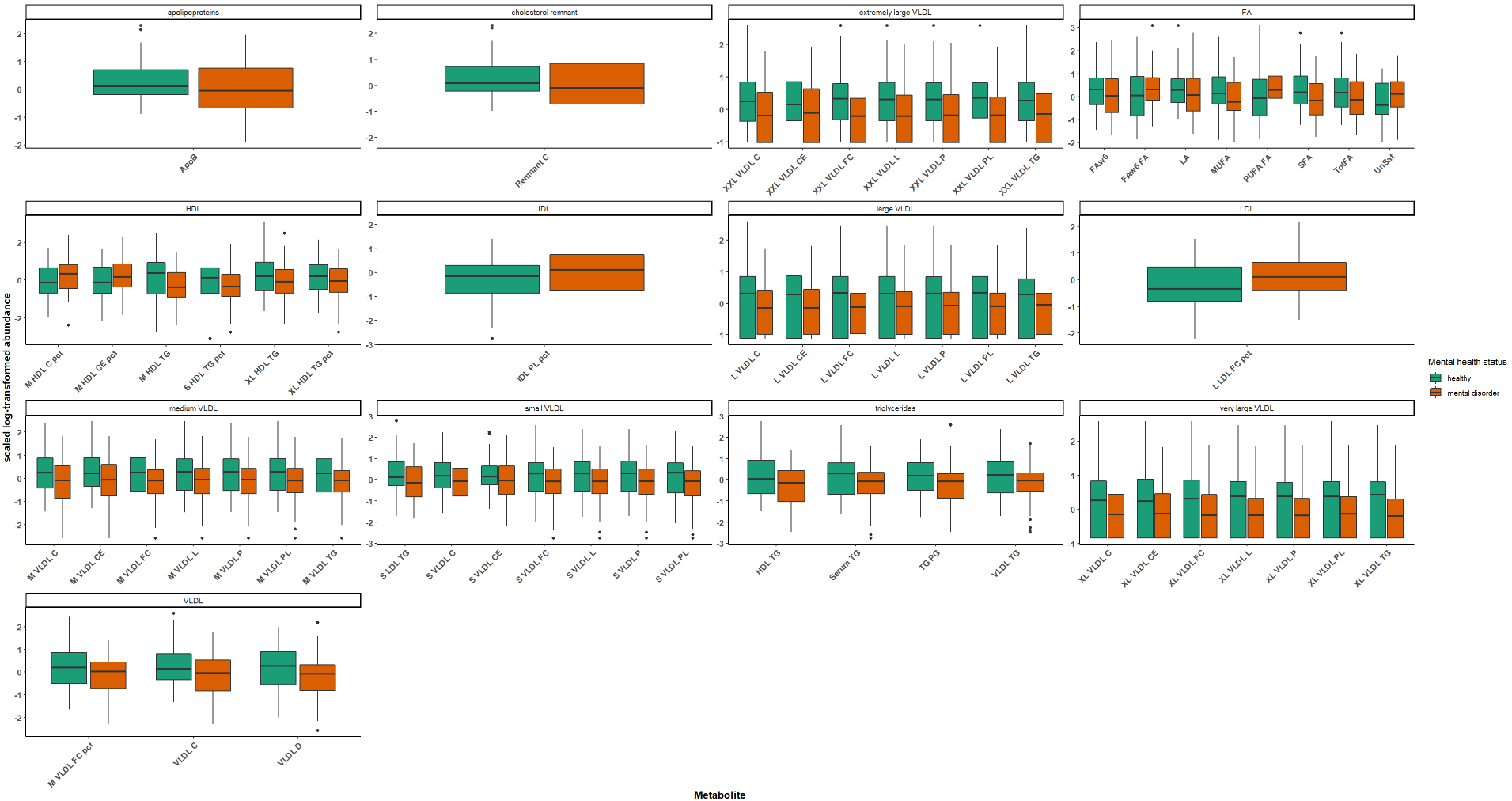


**Supplementary Figure S3** Metabolites differentially abundant in dizygotic discordant twins (paired samples Wilcoxon test, p<0.05). S= small, L = large, M = medium, XL = very large, XXL = extremely large, HDL = high density lipoprotein, LDL= low density lipoprotein, VLDL = very low density lipoprotein, FC = free cholesterol, TG = triglycerides, PL= phospholipids, C = cholesterol, CE = esterified cholesterol, L = total lipids, ApoB = apolipoprotein B, Faw6 = omega-6 fatty acids, LA = linoleic acid, MUFA = mono-unsaturated fatty acids, TotFA = total fatty acids, UnSat = unsaturated fatty acids, SFA = saturated fatty acids, PUFA = poly-unsaturated fatty acids, FAw6 FA = proportion of omega-6 FA of total FA, P = particle numbers.


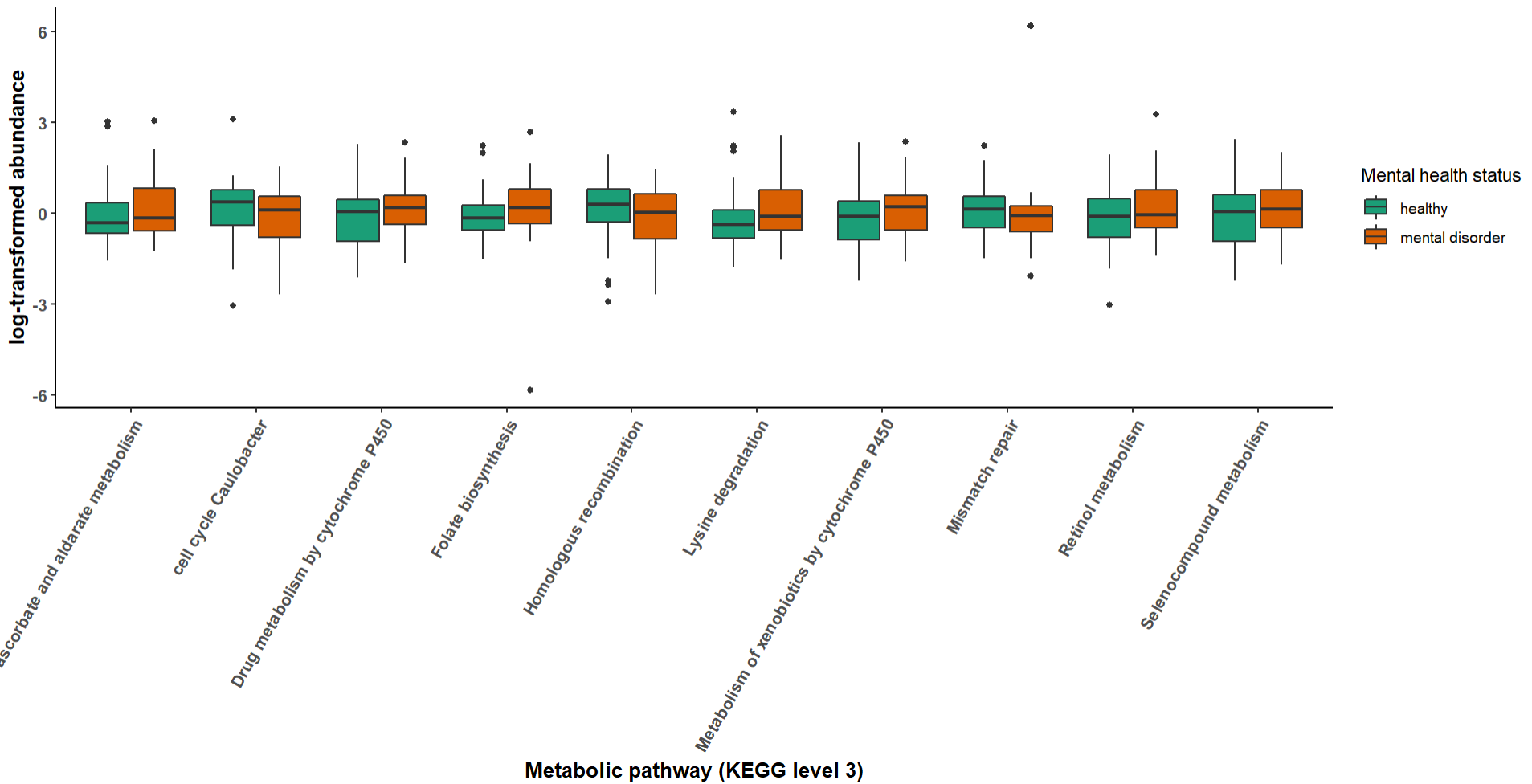


**Supplementary Figure S4** Predicted metabolic pathways (KEGG Level 3) differentially abundant in dizygotic discordant twins (paired samples Wilcoxon test, p-value <0.1).


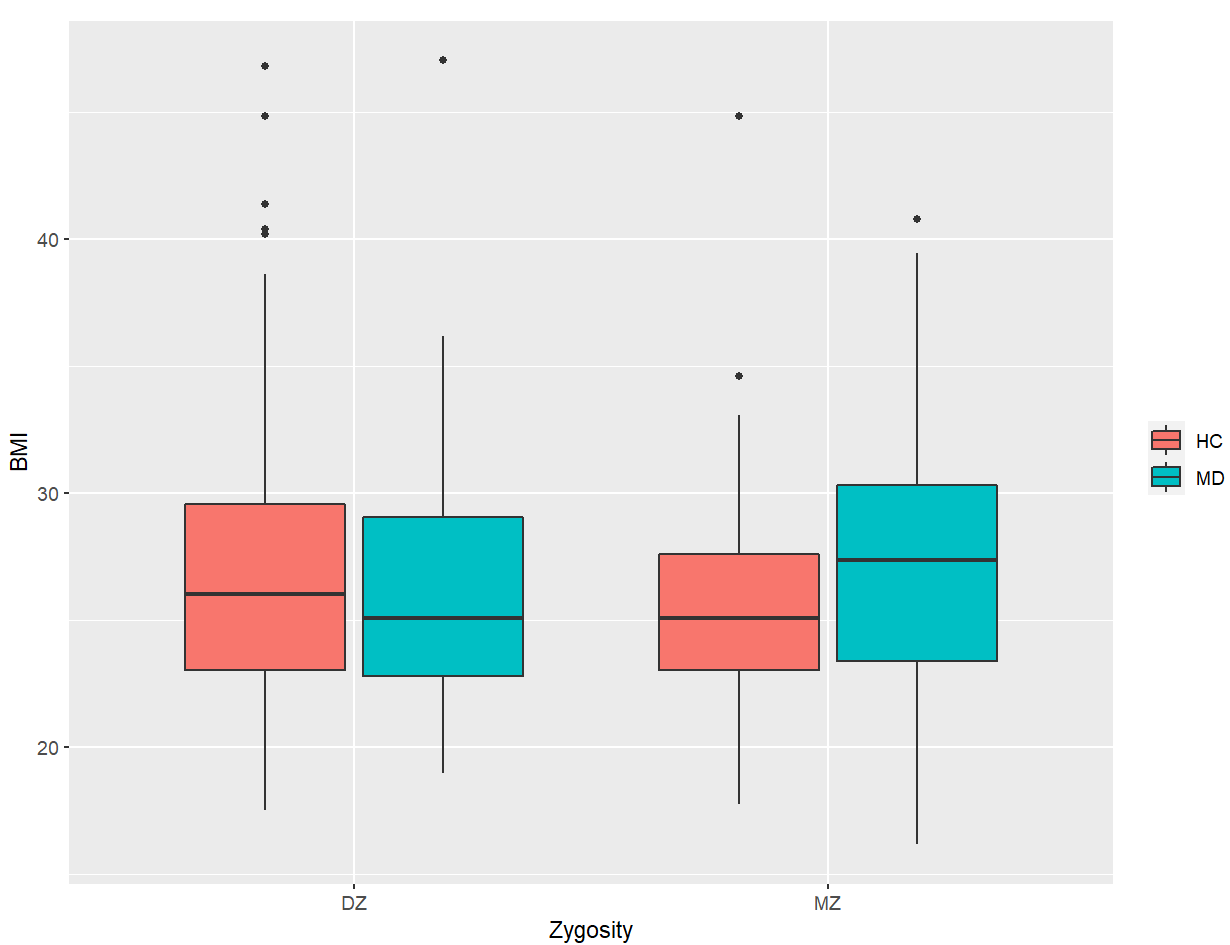


**Supplementary Figure S5** BMI differences between twins with mental disorder (MD) and healthy twins (HC), categorized per zygosity status.

# Supplementary Tables

**Supplementary Table S1** – significant pathways checked against confounders: setup all healthy cotwins (74) vs. all MD-twins (144) (pyramid layer d)) - FDR <0.25

| Predicted pathway | β-estimate fixed effect MD-diagnosis | P-Value | Adjusted p-value |
| --- | --- | --- | --- |
| Ascorbate and aldarate metabolism | 0,309818 | 0,028478 | 0,200222 |
| Retinol metabolism | 0,300583 | 0,027803 | 0,200222 |
| Metabolism of xenobiotics by cytochrome P450 | 0,297234 | 0,02824 | 0,200222 |
| Drug metabolism cytochrome P450 | 0,294879 | 0,030931 | 0,200222 |
| Mismatch repair | -0,24831 | 0,062797 | 0,200222 |
| Homologous recombination | -0,24764 | 0,047737 | 0,200222 |
| Carbapenem biosynthesis | -0,26102 | 0,040643 | 0,200222 |
| Microbial metabolism in diverse environments | 0,257499 | 0,058474 | 0,200222 |
| Glucagon signalling pathway | -0,23322 | 0,066741 | 0,200222 |

**Supplementary Table S2** Significant microbiota checked against confounders: setup all healthy cotwins (74) vs. all MD twins (144) (pyramid layer d) (p<0.05)

| Genus | β-estimate fixed effect MD-diagnosis | P-value | Adjusted p-value |
| --- | --- | --- | --- |
| Parabacteroides | 1,042054567 | 0,002629023 | 0,7327434 |
| Ruminococcaceae UCG 002 | 0,563220468 | 0,008443137 | 0,7327434 |
| Dialister | -1,003701104 | 0,049978898 | 0,7327434 |
| Ruminococcaceae UCG 014 | 0,811428096 | 0,045752946 | 0,7327434 |
| unclassified Christensenellaceae | 0,760848277 | 0,013898812 | 0,7327434 |
| Ruminococcaceae UCG 013 | -0,462784109 | 0,028379426 | 0,7327434 |
| Family XIII AD3011 group | 0,379451262 | 0,038522602 | 0,7327434 |
| Ruminiclostridium 9 | 0,365746623 | 0,039759453 | 0,7327434 |
| Oxalobacter | 0,566653309 | 0,031826666 | 0,7327434 |
| Lachnospiraceae UCG 008 | -0,584924868 | 0,027904822 | 0,7327434 |
| possible genus Sk018 | -0,198847814 | 0,019044744 | 0,7327434 |
| Ezakiella | -0,23599788 | 0,022512989 | 0,7327434 |
| Anaerococcus | -0,120165156 | 0,0396101 | 0,7327434 |
| Sanguibacteroides | -0,258653719 | 0,047803076 | 0,7327434 |
| Lachnospiraceae UCG 006 | -0,175653022 | 0,023443158 | 0,7327434 |
| Clostridium sensu stricto 13 | -0,149520379 | 0,022700564 | 0,7327434 |
| unclassified Subgroup 5 | -0,094403653 | 0,037955648 | 0,7327434 |

**Supplementary Table S3** Significant metabolites checked against confounders in setup all healthy co-twins (74) against all MD-twins (144) (pyramid layer (d)), p<0.05.

| Metabolite | β-estimate fixed effect MD-diagnosis | P-value | Adjusted P-value |
| --- | --- | --- | --- |
| XL_HDL_TG | -0,221118271 | 0,047505667 | 0,4237719 |
| TotPG | -0,210404247 | 0,049462703 | 0,4237719 |
| PC | -0,228470173 | 0,034718491 | 0,4237719 |
| TotCho | -0,225834444 | 0,031342245 | 0,4237719 |
| TotFA | -0,226883765 | 0,036015715 | 0,4237719 |
| LA | -0,223885116 | 0,029966921 | 0,4237719 |
| FAw6 | -0,213371301 | 0,042495614 | 0,4237719 |
| MUFA | -0,203470509 | 0,049980465 | 0,4237719 |
| SFA | -0,247984817 | 0,028201189 | 0,4237719 |

**Supplementary Table S4** Significant microbiota checked against confounders in setup all healthy twins (294) against all MD-twins (144) (pyramid layer (e)), p<0.05.

| Genus | β-estimate fixed effect MD-diagnosis | P-value | Adjusted p-value |
| --- | --- | --- | --- |
| Parabacteroides | 0,49133 | 0,04456 | 0,97742 |
| Ruminococcaceae UCG 002 | 0,50001 | 0,003863 | 0,585314 |
| Bifidobacterium | -0,68292 | 0,00297 | 0,585314 |
| Tyzzerella 4 | -0,46896 | 0,037167 | 0,97742 |
| Ruminiclostridium 9 | 0,333365 | 0,021671 | 0,97742 |
| unclassified Veillonellaceae | -0,33393 | 0,027282 | 0,97742 |
| Lachnospiraceae UCG 008 | -0,4192 | 0,033574 | 0,97742 |
| unclassified DTU 014 | -0,38722 | 0,033577 | 0,97742 |
| Anaerofustis | -0,28986 | 0,028898 | 0,97742 |

**Supplementary Table S5** Significant metabolites checked against confounders in setup all healthy twins (294) against all MD-twins (144) (pyramid layer (e)), p<0.05

| Metabolite | β-estimate fixed effect MD-diagnosis | P-value | Adjusted P-value |
| --- | --- | --- | --- |
| Crea | 0,201783563515827 | 0,0263649857564583 | 0,6568795 |

**Supplementary Table S6** Significant predicted metabolic pathways checked against confounders in setup all healthy co-twins (74) against all MD-twins (144) (pyramid layer (d)), p<0.05

| Prediction | β-estimate fixed effect MD-diagnosis | P-value | Adjusted P-value |
| --- | --- | --- | --- |
| Ascorbate and aldarate metabolism | 0,309818 | 0,028478 | 0,736634 |
| Pentose and glucuronate interconversions | 0,373423 | 0,006415 | 0,736634 |
| Synthesis and degradation of ketone bodies | 0,286446 | 0,024937 | 0,736634 |
| Carbapenem biosynthesis | -0,26102 | 0,040643 | 0,736634 |
| Neomycin kanamycin and gentamicin biosynthesis | -0,27085 | 0,038565 | 0,736634 |
| Glycerophospholipid metabolism | -0,26407 | 0,038923 | 0,736634 |
| Dioxin degradation | 0,306795 | 0,023824 | 0,736634 |
| Propanoate metabolism | 0,366764 | 0,009813 | 0,736634 |
| Retinol metabolism | 0,300583 | 0,027803 | 0,736634 |
| Metabolism of xenobiotics by cytochrome P450 | 0,297234 | 0,02824 | 0,736634 |
| Insect hormone biosynthesis | 0,30072 | 0,029974 | 0,736634 |
| Drug metabolism cytochrome P450 | 0,294879 | 0,030931 | 0,736634 |
| Biosynthesis of unsaturated fatty acids | 0,293928 | 0,026684 | 0,736634 |
| Protein export | -0,34575 | 0,013783 | 0,736634 |
| Homologous recombination | -0,24764 | 0,047737 | 0,736634 |
| Longevity regulating pathway worm | -0,26033 | 0,047703 | 0,736634 |
| Carbohydrate digestion and absorption | -0,32626 | 0,013087 | 0,736634 |

**Supplementary Table S7** Significant metabolic pathways checked against confounders in setup all healthy twins (294) against all MD-twins (144) (pyramid layer (e)) , p<0.05

| Prediction | β-estimate fixed effect MD-diagnosis | P-value | Adjusted P-value |
| --- | --- | --- | --- |
| Pentose and glucuronate interconversions | 0,223991 | 0,03269 | 0,936984 |
| Valine leucine and isoleucine degradation | 0,245217 | 0,019641 | 0,936984 |
| Prodigiosin biosynthesis | 0,218048 | 0,035125 | 0,936984 |
| Dioxin degradation | 0,273516 | 0,008938 | 0,936984 |
| Propanoate metabolism | 0,221849 | 0,035302 | 0,936984 |
| Insect hormone biosynthesis | 0,238629 | 0,021623 | 0,936984 |
| Biosynthesis of unsaturated fatty acids | 0,202524 | 0,048531 | 0,936984 |
| Biosynthesis of vancomycin group antibiotics | 0,209805 | 0,04721 | 0,936984 |

# Supplementary information

### Appendix 1 – Power calculations

We performed power calculations using the full abundance table where we used a Dirichlet-Multinomial distribution to model the statistical parameters for comparing the frequency of all taxa across groups (i.e. healthy controls vs MD). Two types of power calculations were performed. Taxa composition (focus on community composition) and rank abundance distributions (focus on community structure). In taxa composition, we are testing whether the taxa frequencies between cases and controls are similar or far apart, while in the rank abundance distribution we are testing whether differential abundance is different for specific ASVs, and here we can compare, for example, between most abundant ASVs vs most rare ASVs or between species-level taxa.

The taxa composition analysis was used to compare healthy vs MD twins (controls = healthy, cases = MD) (Figure 1), using the function MC.Xdc.statistics from the R package, HMP. To reach **at least 80% of power to detect differences in composition, we need at least 330 healthy samples and 330 MD samples**.

Regarding the rank abundance distribution (RAD) (Figure 2), we followed the approach from Mattiello *et al. (2016), “A web application for sample size and power calculation case-control microbiome studies”*. The ASV table from all TwinsUK individuals was uploaded and defined default parameters were used. Specifically, 1000 Monte Carlo chain (MCMC) replications with 0.05 significance and power were computed. **To reach at least 80% of power to detect differences**  **between diversity we need at least 800 healthy samples and 800 MD samples**. For RAD, the abundances between cases of twins are quite similar, which can be related to **the used assumption that twins shared similar gut environments (maternal microbiome and shared household).** From this it can be seen that large datasets are desired to pick up fine resolution differences between diversities of rare taxa.

An important remark: here, all simulations were performed assuming equal sizes of controls and cases because using this Dirichlet-Multinomial, we are assuming that the distribution of statistical parameters is homogenous given the abundance of data. However, if it is heterogenous, which is the case for our dataset at hand, we will have different statistical parameters. This implies the lack of standardization on microbiome analysis because available datasets are too heterogeneous.

Overall, despite the limitations of statistical power, we conceive the TwinsUK dataset as an unique prospective cohort for novel findings between the gut-brain axis. We believe that our findings alongside previous evidence of the role *Parabacteroides* in mental disorders, can be used as an initial insight for further investigations in general population cohorts. In addition, our results show the need for better protocols and standardization of microbiome-related statistical approaches in biomedical research as well as application in clinical settings.


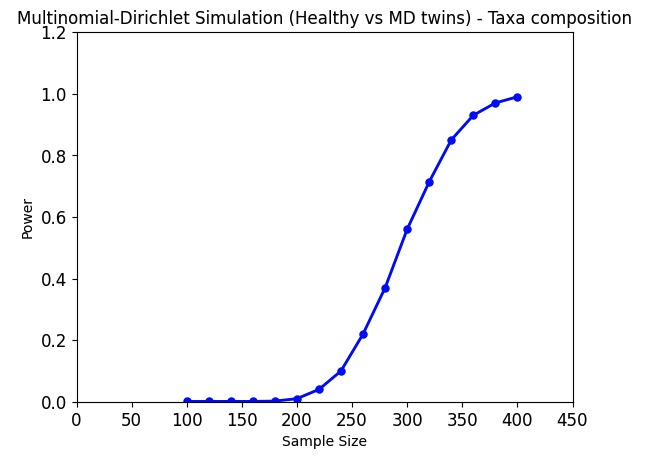


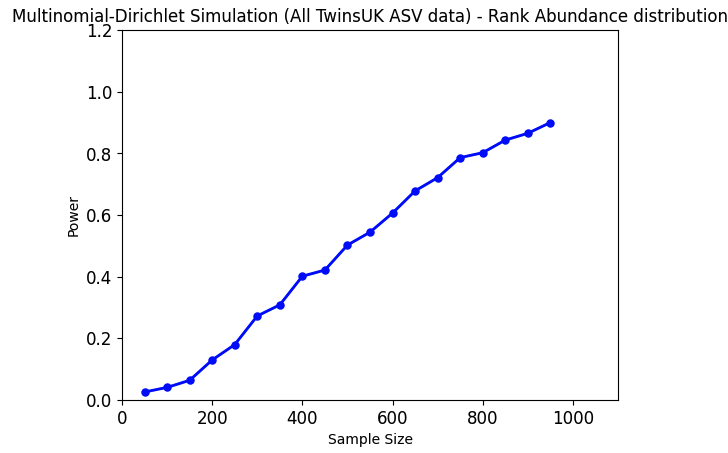

Supplement: Supplementary file 1 — Supplementary Information. [file 41598_2024_53929_MOESM1_ESM.docx]
